# *Baccharis reticularia* DC. and Limonene Nanoemulsions: Promising Larvicidal Agents for *Aedes aegypti* (Diptera: Culicidae) Control

**DOI:** 10.3390/molecules22111990

**Published:** 2017-11-17

**Authors:** Gisele da S. Botas, Rodrigo A. S. Cruz, Fernanda B. de Almeida, Jonatas L. Duarte, Raquel S. Araújo, Raimundo Nonato P. Souto, Ricardo Ferreira, José Carlos T. Carvalho, Marcelo G. Santos, Leandro Rocha, Vera Lúcia P. Pereira, Caio P. Fernandes

**Affiliations:** 1 Walter Mors Institute of Research on Natural Products, Federal University of Rio de Janeiro, Rio de Janeiro 21941-902, Brazil; giselebotas@gmail.com (G.d.S.B.); patrocinio.ufrj@gmail.com (V.L.P.P.); 2 Department of Biological and Health Sciences, Federal University of Amapá, Macapá 68.903-419, Brazil; rodrigo@unifap.br (R.A.S.C.); fb_almeida@id.uff.br (F.B.d.A.); jonatasdlobato@gmail.com (J.L.D.); raquelaraujo_op@yahoo.com.br (R.S.A.); rnpsouto@unifap.br (R.N.P.S.); triato.ricardo@hotmail.com (R.F.); jctcarvalho@gmail.com (J.C.T.C.); 3 Faculty of Teacher Training, University of the State of Rio de Janeiro, São Gonçalo 24435-005, Brazil; marceloguerrasantos@gmail.com; 4 Department of Pharmaceutical Technology, Faculty of Pharmacy, Fluminense Federal University, Niterói 24210-346, Brazil; lean@vm.uff.br

**Keywords:** Asteraceae, early stage fourth-instar larvae, low energy method, scanning electron microscopy

## Abstract

*Baccharis reticularia* DC. is a plant species from the Asteraceae family that is endemic to Brazil. Despite the great importance of *Baccharis* genus, no study has been carried out regarding either the phytochemical composition of *B. reticularia* or the evaluation of its larvicidal potential. Considering the intrinsic immiscibility of essential oils, this study shows larvicidal nanoemulsions containing the *B. reticularia* phytochemically characterized essential oil and its main constituent against *Aedes aegypti*. The major compound found was d-limonene (25.7%). The essential oil inhibited the acetylcholinesterase, one of the main targets of insecticides. The required hydrophile-lipophile balance of both nanoemulsions was 15.0. The mean droplet sizes were around 90.0 nm, and no major alterations were observed after 24 h of preparation for both formulations. After 48 h of treatment, the estimated LC_50_ values were 118.94 μg mL^−1^ and 81.19 μg mL^−1^ for *B. reticularia* essential oil and d-limonene nanoemulsions, respectively. Morphological alterations evidenced by scanning electron micrography were observed on the larvae treated with the d-limonene nanoemulsion. This paper demonstrated a simple and ecofriendly method for obtaining *B. reticularia* essential oil and d-limonene aqueous nanoemulsions by a non-heating and solvent-free method, as promising alternatives for *Aedes aegypti* control.

## 1. Introduction

*Aedes aegypti* (Diptera: Culicidae) is the vector of neglected and emergent tropical diseases. It is the primary dengue and chikungunya vector and, more recently, it was associated to Zika virus outbreak. This is a critical public health problem of international concern due to a possible correlation between infection of pregnant women and neurological disorders, such as microcephaly, in newborns [1]. Several practices of vector control are used against *A. aegypti*, including the mechanical elimination of breeding sites, adulticidal and larvicidal agents [2]. In addition to the removal of breeding sites (also called environmental methods), the mechanical methods may make use of traps. The chemical methods using conventional insecticides may be used either on adults or larvae. However, the problems associated to inducement of resistance is a main issue related to this approach. On the other hand, the biological methods, including those with essential oils have been considered promising [3]. Domestic host breeding sites, such earthenware vases, barrels, cisterns, gutters, cans, tyres and plant saucers, are the main targets for the control. For example, the presence of fertilizers (e.g., NPK) in the water of plant saucers is considered a possible attractant for gravid females [4]. Therefore, the development of alternative larvicides, such those from natural origin, for domestic use should be encouraged.

Asteraceae is considered one of the most representative botanical families among the Angiosperms. In Brazil, around 280 genera and 2075 species can be found [5]. The genus *Baccharis* belongs to this family and has around 178 species distributed in this country [6]. *Baccharis reticularia* DC is endemic and native to Brazil, being found on caatinga, cerrado (Brazilian savanna) and Atlantic forests more specifically, on restinga vegetations (sandy coastal plains) [7]. It is found in open *Clusia* scrub vegetation and open Ericaceae scrub vegetation on the Restinga de Jurubatiba National Park (Rio de Janeiro state, Brazil), being commonly known at this location as alecrim-da-areia (sand-rosemary) due to the fact that it is a high aromatic plant [8]. The antifungal properties of *B. reticularia* have been investigated [9]. However, studies concerning its biological activities are scarce. Moreover, to our knowledge, the chemical constituents of this plant remains unknown, including its volatile constituents.

Essential oils are complex mixtures of volatiles mainly extracted by hydrodistillation or stem distillation, being also able to be extracted by pressing and centrifugation, specifically in the case of citric fruits. They are recognized by several biological properties, such as repellent [10], antimicrobial [11,12], antioxidant [12,13] and larvicidal actions, including against *A. aegypti* larvae [14,15]. Regarding *Baccharis* species, their essential oils were previously reported as antibacterial [16,17,18], repellent [18,19], antiparasitic [16,20], antifungal [16,18] and insecticide [18] agents. However, these complex mixtures have an intrinsic low water miscibility, configuring a technological challenge for aqueous products. Nanoemulsions are disperse systems constituted by two immiscible liquids that are oftenstabilized by one or more surfactants. They have a mean droplet size below 200 nm, kinetic stability, improved bioavailability and enhanced chemical and physical stability of the bioactive compounds [21,22]. In recent years, several studies have been carried out in order to developed new larvicidal formulations using nanotechnology. Nanostructured products prepared with natural herbal oils [23,24,25], including essential oils [26,27,28,29], are considered an excellent eco-friendly option when compared to synthetic pesticides.

However, to our knowledge, no efforts have been carried out to prepare a nanostructured product with the essential oil of *B. reticularia* or to evaluate its larvicidal activity against *A. aegypti*. Thus, the aims of the present study were to elucidate the chemical composition of the essential oil from *B. reticularia* and to prepare and characterize larvicidal nanoemulsions with this natural raw material and its major constituent, using a non-heating and solvent free low energy method, against *A. aegypti* larvae.

## 2. Results

### 2.1. Chemical Composition and Anticholinesterase Activity of the Essential Oil of B. reticularia

The extraction of *B. reticularia* leaves by hydrodistillation yielded 0.30% (*w*/*w*) of an essential oil with slightly green appearance. The phytochemical analysis by gas chromatography with mass spectrometric detection (GC-MS) revealed the presence of 16 identified compounds (Table 1) with a majority of mono- and sesquiterpenes. The relative quantification analysis by gas chromatography with flame ionization detection GC-FID (Table 1) indicated that the most abundant compound was d-limonene (25.7%), a precursor of monoterpene biosynthesis. An unusual component of essential oils was also found (kaurene = 0.7%).

The essential oil from *B. reticularia* was able to inhibit the acetylcholinesterase enzyme with an IC_50_ value of 301.9 μg mL^−1^ (263.2–354.2).

### 2.2. Production and Characterization of B. reticularia Essential Oil and d-Limonene Nanoemulsions

On the day of preparation, most of the nanoemulsions (with hydrophile-lipophile balance–HLB, ranging between 8 and 12) presented a milky aspect which is associated to conventional macroemulsions, in addition to creaming. All the nanoemulsions presented a negative superficial charge. High mean droplet size and polydispersity index were observed mainly for low HLB formulations (Table 2 and Table 3). The best results were obtained with nanoemulsions prepared solely with polysorbate 80 as surfactant (HLB 15), which presented the best maintenance of the physicochemical characteristics after one day of preparation, including a mean droplet size below 200 nm. Considering the observations above, it can be suggested that the required HLB (rHLB) value of both *B. reticularia* essential oil and d-limonene is 15.0.

### 2.3. Larvicidal Assay

**Figure 1:**
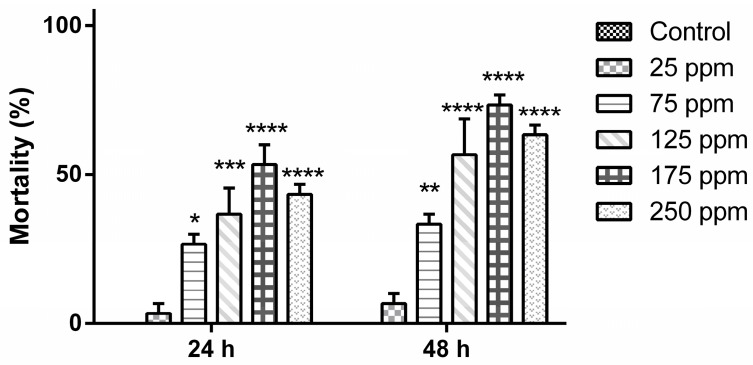
Mortality levels (%) of *Aedes aegypti* (early fourth-instar larvae) after treatment with *Baccharis reticularia* essential oil-based nanoemulsion. Significance: * *p* < 0.05; ** *p* < 0.01; *** *p* < 0.001; **** *p* < 0.0001.

**Figure 2:**
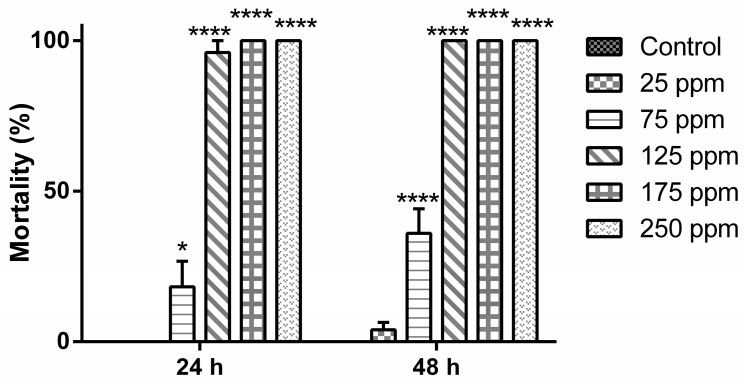
Mortality levels (%) of *Aedes aegypti* (early fourth-instar larvae) after treatment with d-limonene -based nanoemulsion. Significance: * *p* < 0.05; **** *p* < 0.0001.

Considering the best observed parameters, the nanoemulsions of *B. reticularia* essential oil and d-limonene at rHLB = 15 were chosen for further larvicidal assays at different concentrations, as shown in Figure 1 and Figure 2. No mortality level was observed in the control group after 24 h and 48 h, which did not present a statistical significant difference in all periods to the group treated at 25 μg mL^−1^ (*p* > 0.05). No statistical significant difference (*p* > 0.05) in all periods was observed between the group tested at higher concentration (250 μg mL^−1^), when compared to groups treated at 125 and 175 μg mL^−1^. Mortality was time-dependent (*p* < 0.05) in the groups treated with *B. reticularia* nanoemulsion (expressed as essential oil content in water) at 125 μg mL^−1^ (*t*_24h_ = 36.67 ± 15.28%/*t*_48h_ = 56.67 ± 20.82%), 175 μg mL^−1^ (*t*_24h_ = 53.33 ± 11.55%/*t*_48h_ = 73.33 ± 5.77%) and 250 μg mL^−1^ (*t*_24h_ = 43.33 ± 5.77%/*t*_48h_ = 63.33 ± 5.77%).

After 24 h of treatment with *B. reticularia* nanoemulsion, analysis of the data indicated that the percentage of deviance explained by the model was 59.8709 and adjusted percentage was 10.4797. The equation of fitted estimated regression model was y = −1.20057 + 0.00542573x, while *p*-value for the model and *p*-value for the residuals were, respectively, 0.0277 and 0.3547. These results are in agreement with the observed statistical significant differences between the variables and with the idea that the model is not significantly worse than the best possible model at the 95.0% or higher confidence level. The estimated median lethal concentration (LC_50_) and the 90% lethal concentration (LC_90_) values with the lower limit and upper limit are, respectively, 221.273 (151.563–979.895) μg mL^−1^ and 457.472 (299.055–3323.08) μg mL^−1^ (Table 4). After 48 h of treatment, analysis of the data indicated that the percentage of deviance explained by the model was 69.6843 and adjusted percentage was 38.4743. The equation of fitted estimated regression model was y = −1.04355 + 0.00721255x, while *p*-value for the model and *p*-value for the residuals were, respectively, 0.0028 and 0.2741. These results These results are in agreement with the observed statistical significant differences between the variables and with the idea that the model is not significantly worse than the best possible model at the 95.0% or higher confidence level. The estimated LC_50_ and LC_90_ values with the lower limit and upper limit are, respectively, 144.685 (84.1297–228.743) μg mL^−1^ and 322.368 (234.914–748.635) μg mL^−1^ (Table 4).

The main constituent of the *B. reticularia* essential oil, the monoterpene d-limonene, was also subjected for preparation of a larvicidal nanoemulsion. According to Figure 2, no statistical significant difference was observed in the mortality induced by the group treated at 25 μg mL^−1^, in all periods (24 and 48 h) when compared to control group (*p* > 0.05). A significant time-dependent mortality (*p* < 0.0001) was observed only in the group treated at 75 μg mL^−1^ (*t*_24h_ = 20.0 ± 17.32/*t*_48h_ = 36.0 ± 18.17). The highest mortality levels were reached during the first 24 h in the groups treated at 125 μg mL^−1^ (*t*_24h,48h_ = 96.0 ± 8.94%), 175 and 250 μg mL^−1^ (*t*_24h,48h_ = 100%), presenting a statistical significant difference to the control group, 25 and 75 μg mL^−1^ treated groups (*p* < 0.0001).

After 24 h of treatment, analysis of the data indicated that the percentage of deviance explained by the model was 99.9887 and the adjusted percentage was 92.3582. The equation of the fitted estimated regression model was y = −4.74948 + 0.0520471x, while the *p*-value for the model and *p*-value for the residuals were, respectively, 0.0000 and 0.9999. These results corroborate statistical significant differences between the variables and that the model is not significantly worse than the best possible model at the 95.0% or higher confidence level. The estimated LC_50_ and LC_90_ values with the lower limit and upper limit are, respectively, 91.2534 (74.1662–111.616) μg mL^−1^ and 115.876 (99.85–167.279) μg mL^−1^ (Table 4). After 48 h of treatment, analysis of the data indicated that the percentage of deviance explained by the model was 99.2858 and adjusted percentage was 90.0882. The equation of fitted estimated regression model was −2.8997 + 0.0357126x, while the *p*-value for the model and *p*-value for the residuals were, respectively, 0.0000 and 0.9580. These results are in agreement with the observed statistical significant differences between the variables and with the idea that the model is not significantly worse than the best possible model at the 95.0% or higher confidence level. The estimated LC_50_ and LC_90_ values with the lower limit and upper limit are, respectively, 81.1953 (60.1436–102.036) μg mL^−1^ and 117.08 (97.5348–169.639) μg mL^−1^ (Table 4).

### 2.4. A. Aegypti Morphology by Scanning Electron Microscopy

**Figure 3:**
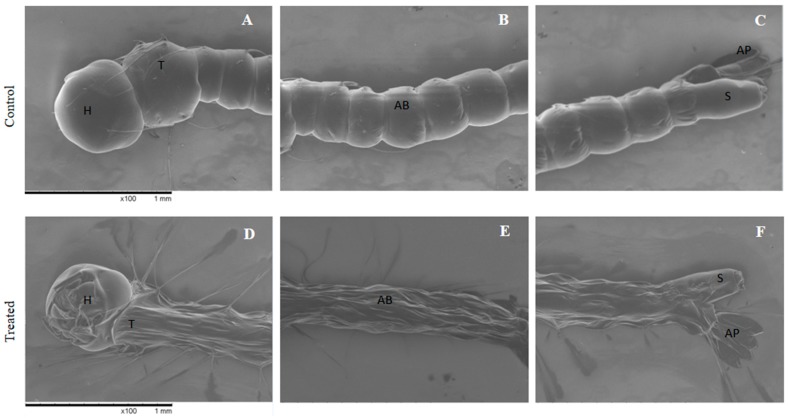
*A. aegypti* larvae morphology by SEM. Control (**A**–**C**) showing no alteration on head (H), thorax (T), abdomen segments (AB), siphon (S) and anal papillae (AP). Larvae treated with nanoemulsion containing d-limonene at 250 ppm (**D**–**F**) showing alterations on head (H), siphon (S) and on cuticles of abdomen (AB) and thorax (T).

The evaluating of *A. aegypti* morphology after exposure to the nanoemulsion prepared with the *B. reticularia* major compound was performed, since highest mortality (100%) was reached with the nanoemulsion prepared with d-limonene, together with a lower LC_50_ value for this nanoemulsion, when compared to the essential oil-based nanoemulsion. Photomicrographs of *A. aegypti* after incubation with the nanoemulsion containing d-limonene at 250 μg mL^−1^ can be seen in Figure 3. The larvae of the control group showed an elongated and vermiform appearance, with the body well defined. The head and the thorax presented a globular aspect, with greater amount of chitin in the cuticles. The abdomen was smooth and flexible, consisting of segments that provided larvae mobility in water. On the other hand, the larvae of the group treated with nanoemulsions containing d-limonene presented a fragile appearance, low resistance, with little mobility and all wrinkled body surface showing alterations on head, thorax, siphon and on cuticles of abdomen. An increase in the number of sows could be also seen (Figure 3D,E).

## 3. Discussion

### 3.1. Chemical Composition and Anticholinesterase Activity of the Essential Oil of B. reticularia

The extraction of essential oil from leaves of *Baccharis reticularia* by hydrodistillation yielded 0.30% (*m*/*m*) which is in accordance with the literature data for the genus, which may range from 0.01 to 1.89% [30,31]. The majority of mono and sesquiterpenes in the essential oil composition is also in accordance with the literature data for the genus *Baccharis* [20,32,33]. The major constituent of the essential oil, d-limonene, is a well-known precursor of monoterpene biosynthesis. d-limonene is known by its antimicrobial activities and also possesses insecticidal properties [34,35]. Although the kaurane-type diterpenes are frequently found on plants of the genus *Baccharis* [33], no reference was found to the presence of these compound on their essential oils. Thus, to our knowledge, this is first report of this type of natural compound as a chemical constituent of essential oils from *Baccharis* species.

The essential oil from *B. reticularia* showed moderate anticholinesterase activity when compared to other oils from the Asteraceae species [36,37]. The inhibition of the AChE is one of proposed mechanisms of insecticide action [38], causing death and paralysis on the insects by blocking neural signal transduction. Essential oils are mixtures of volatile compounds that can be produced by plants as a part of their chemical defense against phytophagous invertebrates mainly by inhibition of this enzyme [39,40]. Despite some ongoing efforts which were carried out to investigate the anticholinesterase activities of extracts and isolated compounds from *Baccharis* spp. [41,42], studies regarding the anticholinesterase potential of the essential oils from this genus still remain scarce. In the present study, the essential oil from *B. reticularia* was able to inhibit the acetylcholinesterase enzyme with an IC_50_ value of 301.9 μg mL^−1^ (263.2–354.2), demonstrating moderate anticholinesterase activity when compared to other oils from Asteraceae species [36,37]. Limonene presented known anticholinesterase activity against some insects, including from *Aedes* genus. Seo and coworkers [43] obtained an IC_50_ value around 130 μg mL^−1^ for the isomer l-limonene against acetylcholinesterase of *Reticulitermes speratus* (Japanese termite). The evaluation of the enantiomers l-limonene and d-limonene against the Culicidae *Aedes albopictus* demonstrated different acetylcholinesterase inhibition of 20% and 40%, respectively when assayed at 1000 μg mL^−1^ [44]. Anticholinesterase assay of the d-limonene against commercial enzymes from *Electrophorus electricus* and butyrylcholinesterase from equine serum were also performed and revealed IC_50_ values of 225.9 ± 1.3 μg mL^−1^ and 456.2 ± 5.6 μg mL^−1^, respectively [45]. Despite the fact that several volatile terpenoids (mono- and sesquiterpenes) show insecticidal activity by inhibitions of AChE [46], some of them may have the activity modulated by the presence of other substances, including those from complex mixtures such as essential oils [47,48]. Based on this preliminary in vitro assay and due to the fact that the essential oil presented a satisfactory IC_50_ value, in accordance with the literature data for its major compound, both essential oil and limonene were used for the preparation of nanoemulsions for evaluation against *A. aegypti* larvae.

### 3.2. Production and Characterization of B. reticularia Essential Oil and d-Limonene Nanoemulsions

Several nanoemulsions with *B. reticularia* essential oil or d-limonene were prepared by using blends of a non-ionic surfactant pair at different ratios, using a low energy and solvent-free method without heating. Despite some studies aiming to generate essential oil-based nanoemulsions focus on high energy methods to generate small size droplets, the utilization of low energy methods that makes use the physicochemical properties of the system are also a good alternative [23,24,25] and should be encouraged, due to the reduced costs of the process. The utilization of non-heating methods is desirable, due to the volatile nature of the compounds of an essential oil [27,28]. Moreover, a solvent-free preparation would lead to less impairment to the environment, being in accordance with a sustainable and eco-friendly approach. rHLB can be predicted based on a series of emulsions prepared with known ratios of a pair of two non-ionic surfactants. It is also a satisfactory strategy to achieve low mean droplet size and its determination has been used to develop larvicidal nanoemulsions [23,24,28].

### 3.3. Larvicidal Assay

The essential oils from some *Baccharis* species were previously subjected to a screening procedure in order to verify their larvicidal activity against late-third/early-fourth *A. aegypti* larvae. At a concentration of 100 μg mL^−1^, following percentages of mortality were observed: *B. dracunculifolia* (55–65%), *B. genistelloides* (20%) *B. pentandlii* (40%) and *B. salicifolia* (40%). Due to different collection places, *B. latifolia* induced 35% of mortality or absence of any activity. However, LC_50_ values of aforementioned essential oils were not estimated [49].

The decrease of 34% on the LC_50_ values of *B. reticularia* nanoemulsion as a function of time observed in this study is in accordance with literature data. Oliveira and coworkers [28] showed 42% of reduction on LC_50_ from 24 to 48 h (371.6 to 213.7 μg mL^−1^) after a larvicidal assay with *Pterodon emarginatus* essential oil-based nanoemulsion against *A. aegypti* larvae. d-limonene nanoemulsions also showed a decrease on its LC_50_ values (11%) from 24 h to 48 h in this study, which is in accordance with the literature. Zahran and coworkers [50] observed a reduction about 11.4% on LC_50_ from 24 h to 48 h from 140 μg mL^−1^ to 124 μg mL^−1^, respectively, after incubation of l-limonene against another Culicidae species (*C. pipens*). Kassir and coworkers [51] observed a 20% LC_50_ reduction from 24 to 48 h (from 53.8 to 32.52 μg mL^−1^) after incubation of pure limonene with *Culex quinquefasciatus*. The enhancement of activity may be associated to gradative release of the larvicidal compounds from nanostructure systems as nanoemulsions [52]. Further studies aiming to correlate the release of compounds with mortality as function of time should be performed to better understanding of the phenomena involved in the larvicidal action of nanoemulsions, including those based on *B. reticularia*.

The estimated LC_50_ value for d-limonene nanoemulsions obtained in this study is close to the one reported d-limonene non-nanoemulsified against fourth-instar larvae of *A. aegypti* (71.9 μg mL^−1^) [53]. The highest mortality levels were reached during the first 24 h on the groups treated at 125, 175 and 250 μg mL^−1^. This data is in accordance with Pavela and coworkers [54] that found 100% of mortality induced by d-limonene on *Culex quinquefasciatus* larvae at 250 μg mL^−1^.

### 3.4. A. Aegypti Morphology by Scanning Electron Microscopy

The observed morphological alterations in treated *A. aegypti* larvae are in accordance with previous works [25] and may affect larvae development and motility contributing to the observed high mortality. For example, the increase in the number of sows can hamper the exoskeleton exchange process, as seen by Borges and coworkers [55]. However, other factors can contribute for larvicidal activity on mosquito larvae, such as the damage to the digestive tube which is associated to anti-feedant behavior [56].

## 4. Materials and Methods

### 4.1. Chemicals

Polysorbate 80 and sorbitan monooleate were obtained from Praid (São Roque, SP, Brazil). *n*-alkanes (C_7_–C_40_), limonene, acetylthiocholine iodide (ATCI) and 5,5-dithiobis-2-nitrobenzoic acid (DTNB) were purchased from Sigma-Aldrich (St. Louis, MO, USA). Distilled water was used for general procedures. All chemicals were of analytical grade.

### 4.2. Plant Material

The leaves of *B. reticularia* (400 g) were collected at Restinga de Jurubatiba National Park, Rio de Janeiro State, Brazil (22°14.105′ S, 41°35.822′ W). The identification was performed by the Botanist Dr. Marcelo Guerra Santos, and voucher specimen of *B. reticularia* was deposited at the herbarium of the Faculdade de Formação de Professores (Universidade do Estado do Rio de Janeiro, São Gonçalo, RJ, Brazil) under the register number RFFP 2097. The nomenclatural update was realized in Lista de Espécies da Flora do Brazil (http://floradobrasil.jbrj.gov.br), and The Plant List: A Working List of All Plant Species (http://www.theplantlist.org/).

### 4.3. Gas-Chromatographic Conditions and Identification of Chemical Constituents

The essential oil was analyzed by a GC-MS-QP2010 gas chromatograph equipped with a mass spectrometer using electron ionization (Shimadzu, Barueri, SP, Brazil). The GC conditions were as follows: Injector temperature, 260 °C; detector temperature, 290 °C; carrier gas (Helium), flow rate 1 mL min^−1^, and split injection with split ratio 1:40. Oven temperature was initially 60 °C and then raised to 290 °C at a rate of 3 °C min^−1^. The sample was diluted with n-hexane (1:100, *v*/*v*) and injected on a ZB-5 column (i.d. = 0.25 mm, length 30 m, film thickness = 0.25 μm). The MS conditions were voltage, 70 eV, and scan rate; 1 scan s^−1^. The retention index was calculated by the interpolation of each substance retention time and the retention time of a mixture of aliphatic hydrocarbons analyzed in the same conditions [57]. The identification of substances was performed by comparison of their retention index and mass spectra with those reported in the literature [58]. MS fragmentation pattern of compounds was also checked with NIST (National Institute of Standards and Technology) mass spectra libraries. Quantitative analysis of the chemical constituents was performed by GC-FID (Shimadzu, Barueri, SP, Brazil), under the same conditions of GC-MS analysis and percentages obtained by GC-FID were performed by peak area normalization method.

### 4.4. Quantitative Determination of B. reticularia Essential Oil Anticholinesterase Activity

Acetylcholinesterase (AChE) activity assay was performed using a method that uses acetylthiocholine iodide as substrate [59], with some modifications. 340 μL of test solution (1.25 mg mL^−1^ in MeOH), 1660 μL of 0.1 mM sodium phosphate buffer (pH 7.5) and 200 μL of AChE solution (30 mU/mL, sodium phosphate buffer 0.1 M pH 7.5) were mixed and incubated for 10 min at 25 °C. The reaction started with the addition of 1000 μL of 5,5′-Dithiobis(2-nitrobenzoic acid) (DTNB, 0.68 mM) and 200 μL of acetylthiocholine iodide (17 mM). The hydrolysis of acetylthiocholine iodide was monitored by the formation of the yellow 5-thio-2-nitrobenzoate anion as a result of the reaction of DTNB with thiocholine at 412 nm. The IC_50_ values (the concentration of test compounds that inhibit the hydrolysis of substrates by 50%) were estimated by linear regression of the natural log of concentration of essential oil versus percentage of remaining enzyme activity in the presence of essential oil and then solving the resulting equation for a 50% remaining activity [60]. The experiments were carried out in triplicate. Physostigmine was used as positive control. One unit of enzyme activity was defined as the amount of enzyme that produced 1 μmol of 5-thio-2-nitrobenzoate anion in 1 min under the conditions defined.

### 4.5. Determination of Required Hydrophile-Lipophile Balance (rHLB) of B. reticularia Essential Oil and Its Major Compound

Two non-ionic surfactants with low and high hydrophile-lipophile balance value (HLB) were blended together in order to achieve a wide range of HLB values (8.0–15.0). rHLB value of each blend was calculated as follows: rHLB = [(HLBsm × mSm) + (HLBp80 × mP80)]/(mSm + mP80), where HLBsm is the HLB of sorbitan monooleate, HLBp80 is the HLB of polysorbate 80, mSm is the mass (g) of sorbitan monooleate and mP80 is the mass of polysorbate 80. rHLB value of the *B. reticularia* essential oil and its major compound were determined as the HLB value of single surfactant or surfactant blend that was able to induce formation of most stable nanoemulsion.

### 4.6. Nanoemulsification

The nanoemulsions were prepared according to a non-heating and low energy method [61]. The *B. reticularia* essential oil and surfactant(s) were pooled together and homogenized for 30 min. Then, distilled water was added dropwise and the system was submitted to magnetic stirring for 1 h. The final concentration of *B. reticularia* essential oil was 2500 μg mL^−1^ and surfactant to oil ratio (SOR) was 1:1. This same procedure was used for the preparation of a nanoemulsion with the main constituent of *B. reticularia* essential oil.

### 4.7. Particle Size Distribution and Zeta Potential Measurements

Photon correlation spectroscopy (PCS) analysis was carried out using a Zetasizer Nano ZS, (Malvern Instruments, Malvern, UK) equipped with a 10 mW “red” laser (X = 632.8 nm). Samples were measured at a 90° scattering detector angle immediately after preparation (Day 0) and after 24 h (Day 1). The nanoemulsions were diluted with deionized water (1:25, *v*/*v*) for analysis. The measurements of droplet size, polydispersity index and zeta potential were performed in triplicate. Data was expressed as the mean ± standard deviation.

### 4.8. Larvicidal Activity

*Aedes aegypti* larvae were obtained from the Arthropoda Laboratory (Universidade Federal do Amapá, Macapá, AP, Brazil). Biological assay was performed under controlled conditions, being early fourth-instar larvae kept at 25 ± 2 °C, relative humidity of 75 ± 5% and a 12 h light-dark cycle. The experimental evaluation was performed according to World Health Organization protocol [62] with some modifications. All the experiments were performed in triplicate with 10 early stage fourth-instar larvae in each sample. *B. reticularia* essential oil and d-limonene nanoemulsions were diluted separately in distilled water at 25, 75, 125, 175 and 250 μg mL^−1^ (concentration expressed as essential oil or major compound content on aqueous media). The control group was constituted by deionized water. Mortality levels were recorded after 24 and 48 h of exposure.

### 4.9. Morphological Aedes Aegypti Larvae Study

The morphology of larvae was obtained according to Oliveira and coworkers [25]. Briefly, the larvae were incubated with the nanoemulsion containing the major compound at 250 μg mL^−1^, since it induced the highest mortality. After, they were fixed on ethanol 70% and evaluated by scanning electron microscopy under low vacuum using a Tabletop Microscope TM3030Plus (Hitachi, Ibaraki, Japan).

### 4.10. Statistical Analysis

Analysis of variance (two-way ANOVA) followed by Tukey’s test or Bonferroni’s test and linear regression for IC_50_ determination were conducted using the Software GraphPad Prism 6.0 (San Diego, CA, USA). Differences were considered significant when *p* < 0.05. Probit analysis was performed with 95% confidence interval for LC_50_ and LC_90_ determination using the software Statgraphics Centurion XV version 15.2.11 (Statpoint Technologies, The Plains, VA, USA).

## 5. Conclusions

Few studies about preparation of nanoemulsions by low energy methods with essential oils are available when compared to high-energy methods. In addition to the successful preparation of nanoemulsions with *B. reticularia* essential oil and d-limonene by a titration non-heating and solvent-free method, we showed the larvicidal potential of these nanostructured systems against *A. aegypti*, the main vector of the dengue, zika and chikungunya viruses. The facility of nanoemulsion preparation using an ecofriendly approach and the larvicidal activity indicate great perspectives for the further utilization of these raw materials for nanophytoproducts, which are potentially useful to control the mosquito vector by dispersing low water soluble compounds in aqueous media through innovative nanoemulsions.

## Figures and Tables

**Table 1:** Chemical constituents of the essential oil from *B. reticularia* and their relative abundance.

RI	Compound	%
937	α-Pinene	7.3
976	Sabinene	0.9
981	β-Pinene	8.4
991	β-Myrcene	8.5
1026	*p*-Cymene	0.5
1034	d-limonene	25.7
1177	Terpin-4-ol	0.5
1389	β-Elemene	1.2
1418	(*E*)-caryophyllene	24.6
1481	d-Germacrene	1.7
1494	Bicyclogermacrene	11.3
1518	δ-Cadinene	1.1
1580	Spathulenol	3.2
1588	Globulol	0.8
1596	Viridiflorol	0.8
2047	Kaurene	0.7
Total of monoterpenes		51.8
Total of sesquiterpenes		44.7
Total of diterpenes		0.7
Total of identified compounds		97.2

RI: retention index.

**Table 2:** Physicochemical characterization of nanoemulsions containing *B. reticularia* essential oil.

HLB	Size ± SD (nm)	Pdi ± SD	Zeta ± SD (mV)	Size ± SD (nm)	Pdi ± SD	Zeta ± SD (mV)
15	92.9 ± 0.4	0.412 ± 0.009	−20.4 ± 0.6	94.5 ± 1.9	0.382 ± 0.048	−21.5 ± 1.4
14	162.3 ±1.4	0.392 ± 0.007	−26.0 ± 0.5	159.1 ± 2.2	0.416 ± 0.029	−26.6 ± 0.6
13	304.5 ± 134.2	0.493 ± 0.027	−32.5 ± 0.9	208.1 ± 11.9	0.497 ± 0.030	−36.7 ± 3.7
12	814.6 ± 943.0	0.714 ± 0.224	−32.3 ± 0.6	371.8 ± 254.7	0.581 ± 0.032	−36.0 ± 1.0
11	793.3 ± 687.4	0.661 ± 0.299	−34.8 ± 0.6	434.8 ± 242.6	0.691 ± 0.183	−39.6 ± 0.5
10	1224.0 ± 568.9	0.846 ± 0.144	−36.3 ± 0.6	1131.0 ± 649.7	0.856 ± 0.131	−42.7 ± 0.3
9	1157.0 ± 965.5	0.802 ± 0.178	−40.5 ± 1.7	1231.0 ± 784.8	0.886 ± 0.099	−45.8 ± 2.9
8	938.6 ± 553.6	0.722 ± 0.132	−45.1 ± 1.8	1208.0 ± 1035.0	0.772 ± 0.197	−50.1 ± 1.2

Pdi: polydispersity index; SD: standard deviation.

**Table 3:** Physicochemical characterization of nanoemulsions containing d-limonene.

HLB	Size (nm)	Pdi	Zeta (mV)	Size (nm)	Pdi	Zeta (mV)
15	136.0 ± 2.9	0.728 ± 0.030	−15.4 ± 0.4	138.0 ± 1.0	0.453 ± 0.006	−18.3 ± 0.3
14	154 ± 3.0	0.516 ± 0.031	−15.0 ± 0.4	172.0 ± 0.6	0.528 ± 0.005	−20.8 ± 0.5
13	177.5 ± 3.86	0.471 ± 0.015	−24.5 ± 0.6	165.8 ± 0.8	0.462 ± 0.013	−24.1 ± 0.6
12	162 ± 0.902	0.627 ± 0.040	−29.6 ± 0.5	198.0 ± 14	0.655 ± 0.008	−28.6 ± 0.7
11	292 ± 16.91	0.690 ± 0.029	−37.1 ± 1.4	193.9 ± 45	0.655 ± 0.085	−36.8 ± 0.5
10	624.9 ± 80.51	0.869 ± 0.043	−45.4 ± 0.4	409.6 ± 71	0.762 ± 0.050	−45.4 ± 0.0

HLB: hydrophile-lipophile balance; Pdi: polydispersity index; SD: standard deviation.

**Table 4:** Larvicidal activity of nanoemulsions of *B. reticularia* essential oil and d-limonene.

Nanoemulsion	24 h		48 h	
	LC_50_	LC_90_	LC_50_	LC_90_
*B. reticularia*	221.273 (151.563–979.895)	457.472 (299.055–3323.08)	144.685 (84.1297–228.743)	322.368 (234.914–748.635)
d-limonene	91.2534 (74.1662–111.616)	115.876 (99.85–167.279)	81.1953 (60.1436–102.036)	117.08 (97.5348–169.639)

LC_50_ and LC_90_ expressed in μg mL^−1^ (lower limit–upper limit).
